# Hookworm infection, anaemia and genetic variability of the New Zealand sea lion

**DOI:** 10.1098/rspb.2009.1001

**Published:** 2009-07-15

**Authors:** Karina Acevedo-Whitehouse, Laura Petetti, Padraig Duignan, Aurelie Castinel

**Affiliations:** 1 Institute of Zoology, Regent's Park, London NW1 4RY, UK; 2 Institute for Natural Resources, Massey University, Palmerston North, New Zealand; 3 Ministry of Agriculture and Forestry, Wellington 6011, New Zealand

**Keywords:** anaemia, haemostasis, heterozygosity, hookworms, inbreeding, New Zealand sea lion

## Abstract

Hookworms are intestinal blood-feeding nematodes that parasitize and cause high levels of mortality in a wide range of mammals, including otariid pinnipeds. Recently, an empirical study showed that inbreeding (assessed by individual measures of multi-locus heterozygosity) is associated with hookworm-related mortality of California sea lions. If inbreeding increases susceptibility to hookworms, effects would expectedly be stronger in small, fragmented populations. We tested this assumption in the New Zealand sea lion, a threatened otariid that has low levels of genetic variability and high hookworm infection rates. Using a panel of 22 microsatellites, we found that average allelic diversity (5.9) and mean heterozygosity (0.72) were higher than expected for a small population with restricted breeding, and we found no evidence of an association between genetic variability and hookworm resistance. However, similar to what was observed for the California sea lion, homozygosity at a single locus explained the occurrence of anaemia and thrombocytopenia in hookworm-infected pups (generalized linear model, *F* = 11.81, *p* < 0.001) and the effect was apparently driven by a particular allele (odds ratio = 34.95%; CI: 7.12–162.41; *p* < 0.00001). Our study offers further evidence that these haematophagus parasites exert selective pressure on otariid blood-clotting processes.

## 1. Introduction

Natural populations commonly harbour substantial variation in their ability to resist infection. Such variation is largely driven by genetic factors (Stear & Wakelin 1998). Low levels of heterozygosity have been related to impaired immunocompetence (e.g. Hawley *et al*. 2005), higher pathogen loads (e.g. Luikart *et al*. 2008), and greater susceptibility to infections and higher disease severity (e.g. Acevedo-Whitehouse *et al*. 2003). Correlations between heterozygosity and various aspects of fitness (HFCs) appear to be widespread and common in wildlife and have been generally interpreted as resulting from inbreeding depression (i.e. genome-wide reduction in heterozygosity; Crow 1948). However, the mechanisms underlying HFCs still remain contentious (Hansson & Westerberg 2002). While multi-locus heterozygosity may accurately reflect inbreeding in small fragmented populations or in highly territorial species with polygamous mating systems (Balloux *et al*. 2004), HFCs are most likely to be caused by ‘associative overdominance’ (Frydenberg 1963); that is, by a subset of the loci used for genotyping being linked to functional loci showing heterozygote advantage (Hansson *et al*. 2004). As yet, relatively few wildlife studies have investigated the importance of single-locus effects in disease-related HFCs, but evidence that these may be prevalent is slowly accumulating (e.g. Acevedo-Whitehouse *et al*. 2005; Amos & Acevedo-Whitehouse 2009), suggesting that HFCs might be useful to identify regions undergoing parasite-driven selection, as has been well documented for human malaria (reviewed in Verra *et al*. 2009).

A recent study investigated the relative importance of both mechanisms for susceptibility to hookworms (*Uncinaria* spp.) in California sea lions (Acevedo-Whitehouse *et al*. 2006). Hookworms are haematophagus nematodes that affect a wide range of mammals, including humans and various wild species, causing intestinal haemorrhage, severe anaemia and protein malnutrition (Hotez *et al*. 2004). Acevedo-Whitehouse *et al*. (2006) found that while inbreeding was a good predictor of hookworm-induced lesions and mortality, anaemia was explained by homozygosity at a single locus, suggesting that hookworms may drive selection on blood-clotting mechanisms (haemostasis) in this species.

Hookworms are likely to exert strong selective pressure as they infect large numbers of individuals and can cause high mortality rates. For instance, hookworm infections were found in 100 per cent of California sea lion pups examined at San Miguel Island (USA) in various years (Lyons *et al*. 2001, 2005) and caused over 70 per cent of the total pup mortality (Lyons *et al*. 2005). Hookworms infect several otariid species, including the New Zealand sea lion (*Phocarctos hookeri*; hereafter NZSL), considered one of the rarest and most locally endemic otariid species (Campbell *et al*. 2006). Hookworms are transferred directly to the newborn pup's gut via infected maternal milk. They attach to the intestinal mucosa and feed on blood, causing anaemia and haemorrhagic enteritis (Castinel *et al*. 2007*a*,*b*; Spraker *et al*. 2007). Hookworm burdens are highest during the first three or four months and then gradually decrease, being undetectable in pups after 12 months (Lyons *et al*. 2003). This suggests that otariids acquire immunity against hookworms some time before weaning (Acevedo-Whitehouse *et al*. 2006).

If low genetic variability poses a disadvantage for hookworm resistance and increases the probability of hookworm-induced mortality, effects could be important for the NZSL, which has an extremely restricted breeding distribution and a small and declining population size (less than 10 000 adults; Campbell *et al*. 2006). Furthermore, considering the parasite's wide host range and pathogenesis, it is possible that hookworms drive selection on platelet (PLT) function or blood-clotting across different host species. We tested these assumptions in NZSL pups by examining whether (i) relatively less heterozygous (i.e. more inbred) individuals are more susceptible to hookworm infection and (ii) occurrence of hookworm-related anaemia is explained by associative overdominance, as observed for California sea lions (Acevedo-Whitehouse *et al*. 2006). We genotyped 22 microsatellites in 39 NZSL pups from a single colony. Hookworm counts, full pathology reports (of dead pups, *n* = 25) and blood parameters (of live pups, *n* = 14) were available. We were unable to detect an association between levels of genetic variability and hookworm resistance. Similar to what was observed for California sea lions, homozygosity at a single locus explained the occurrence of anaemia and thrombocytopenia, further strengthening the suggestion that hookworms exert selective pressure on haemostasis.

## 2. Material and methods

### (a) Study population and sampling

As part of an NZSL mortality survey conducted between the 2001 and 2005 breeding seasons, pups found dead on Sandy Bay beach, Enderby Island (50°30′ S, 166°17′ E) were examined following a standard protocol. Sandy Bay beach is the species' second-largest breeding colony (Robertson *et al*. 2006), with approximately 400 pups born annually and mortality rates of around 14 per cent (Castinel *et al*. 2007*b*). We selected 25 freshly dead hookworm-infected pups (three to eight weeks of age), for which histopathology and parasitological data were available. Hookworm infection intensity had been determined for each of the selected pups (Castinel *et al*. 2007*a*). A qualitative assessment of status of anaemia (yes/no) was made at necropsy based on carcass pallor and blood consistency.

An additional set of skin biopsies and blood samples was collected from 14 live pups (two to eight weeks of age). Haematological parameters—erythrocyte volume fraction (haematocrit, HCT), leukocytes (white cell count, WCT) and thrombocytes (PLT)—were quantified for these pups as they are relevant to hookworm infections (Hotez *et al*. 2004). For live pups, status of anaemia (yes/no) was determined by clinical examination (capillary refill time greater than 2 s, pallor of mucous membranes) and corroborated by the levels of HCT (less than or equal to 30%; Bossart *et al*. 2001).

### (b) Microsatellite genotyping

DNA was extracted from each sample using a Chelex-proteinase K digestion (Acevedo-Whitehouse 2004) followed by purification using DNeasy spin columns (Qiagen, UK), according to the manufacturer's instructions. When the quality (*A*_260_/*A*_280_ ratio) of a sample was lower than 1.65, DNA was re-extracted to avoid contamination with protein or RNA, which could interfere with the analyses.

We PCR-amplified 22 pinniped microsatellite loci (table S1, electronic supplementary material). Multiple reactions were performed using four to five pairs of fluorescent end-labelled primers, grouped according to optimized annealing temperatures and expected product sizes. Amplified products were run on an ABI 3100 automatic sequencer (Applied Biosystems, UK). Alleles were inspected manually and were scored using GeneMapper v. 3.7 (Applied Biosystems).

Homozygous individuals were genotyped twice to reduce errors owing to allele dropout. Allelic disequilibrium and non-amplifying alleles were tested by investigating deviation from Hardy–Weinberg equilibrium (HWE) using GENEPOP v. 3.3 (Raymond & Rousset 1995).

### (c) Calculation of individual levels of genetic variability

We calculated three surrogate measures of inbreeding: parental relatedness (IR; Amos *et al*. 2001), homozygosity by loci (HL; Aparicio *et al*. 2006) and standardized mean (*d*^2^; Coulson *et al*. 1998; Hedrick *et al*. 2001). We calculated all three measures because IR was found to be a highly sensitive measure for detecting differences in resistance to hookworms in California sea lions (Acevedo-Whitehouse *et al*. 2006). This could partly be explained because it weighs common homozygous alleles less than rare ones, and these could plausibly be linked to recessive harmful gene variants (Amos *et al*. 2001). However, a more recent measure, HL, weighs the contribution of each locus to homozygosity, according to their allelic variability, and thus will hypothetically improve estimates of heterozygosity (Aparicio *et al*. 2006). Standardized mean, *d*^2^, assumes that microsatellites mutate mainly by stepwise changes in the number of repeat units and thus is theoretically a useful measure of inbreeding related to more distant events in population lineages (Hedrick *et al*. 2001).

HL was calculated in STORM v. 1.0, a program written in C by T. Frasier (http://web.nrdpfc.ca/bios/timsoft.htm). IR and *d*^2^ were calculated using IRmacroN, an Excel macro written in Visual Basic by W. Amos (www.zoo.cam.ac.uk/zoostaff/amos/#Computerprograms).

### (d) Statistical analyses

The association between the measures of inbreeding and survival was explored in a generalized linear model (GLM) controlling for age. Hookworm count intensity parameters (skew, mean, median and exact confidence intervals) were calculated in Quantitative Parasitology v. 3.0 (Rozsa *et al*. 2000). A linear regression was used to investigate the relationship between measures of inbreeding and hookworm burden. The effects of inbreeding on hookworm-related mortality and on anaemia were independently evaluated using a series of GLMs accounting for age. The response variables were independently defined as a binary response in each model (no, 0; yes,1) and modelled using a binomial error structure. All analyses were performed in R v. 2.5.0. To account for multiple testing, we conducted sequential Bonferroni procedures.

### (e) Detection of single-locus effects

The presence of inbred individuals may be indicated by a tendency for heterozygosity to be correlated among loci (Balloux *et al*. 2004). To test for this correlation, the 22 loci included in our study were randomly divided into two equal subsets of 11 markers, each of which was used to calculate inbreeding (IR and HL) for each pup. This procedure was repeated 100 times to obtain the mean and standard deviation of the correlation coefficient among loci. GLMs were constructed to determine the contribution of each microsatellite for different aspects of hookworm infection, which would expectedly arise owing to physical linkage with a gene experiencing balancing selection for the trait examined (Ohta 1971; Hansson *et al*. 2004). The importance of the genotype for the response variable was assessed by calculating the Peto odds ratio (OR; Yusuf *et al*. 1985). We assumed a null hypothesis that genotype had no effect on the outcome, so the difference between the observed and expected values would have zero difference and variance, allowing us to include values of zero without generating infinity (Deeks *et al*. 1998). Significance was established using Fisher's exact tests. Calculations were conducted within the freeware OR calculator (www.hutchon.net/ConfidORnulhypo.htm).

## 3. Results

All microsatellites included in the study were relatively polymorphic in the NZSL, with two alleles found at the least variable locus (ZcCgDh1.8) and 11 alleles found at the most variable locus (ZcwA07). For all loci combined, the average number of alleles was 5.9. Average heterozygosity was 0.72, ranging from 0.43 to 1. Genotype frequencies did not differ significantly from HWE expectations (table S1, electronic supplementary material), except for locus Lw10, which had a significant excess of heterozygotes (*p* = 0.001) and was removed from subsequent calculations.

Values for all the three estimates of inbreeding had a normal distribution (Shapiro–Wilk normality test; *p* > 0.45) and were significantly correlated (IR and HL, *r* = 0.97, *p* < 0.001; standardized mean *d*^2^ and IR, *r* = −0.48, *p* < 0.01). None of the measures had a significant effect on pup survival (*p* > 0.1 for all tests). Age was not a significant variable in any of the three models and was subsequently removed from calculations. Only three pups had moderately high values of parental relatedness (IR = 0.29, 0.10 and 0.10).

Hookworm counts fitted a negative binomial distribution (small sample skewness measure = −0.84 with respect to the negative binomial, *p* < 0.05; see fig. S1 in the electronic supplementary material). Hookworm intensity mean was 1820 (range 57–4080) and intensity median was 1500 (97.1% exact CI: 1000–2000). Variance/mean ratio was 902.93. None of the estimates of inbreeding was significantly related to hookworm burden (*p* > 0.05 for all estimates).

Although all pups were infected, hookworms caused the death of only 16 (64%) pups. The rest died owing to drowning, trauma or pyogenic infections secondary to trauma. Independent testing of IR, HL and *d*^2^ as predictors of hookworm-related death did not reveal any significant effect (*p* > 0.1 in all models). Eleven of the dead pups (44%) presented evidence of anaemia owing to blood loss, and four of the live pups (29%) were anaemic. Mean measures of inbreeding did not vary between anaemic and non-anaemic pups (*p* > 0.05), although the difference in mean values did go in the direction expected if heterozygosity decreases blood loss during hookworm infection, anaemic pups being on average slightly more homozygous than pups that did not reveal anaemia.

**Figure 1.**
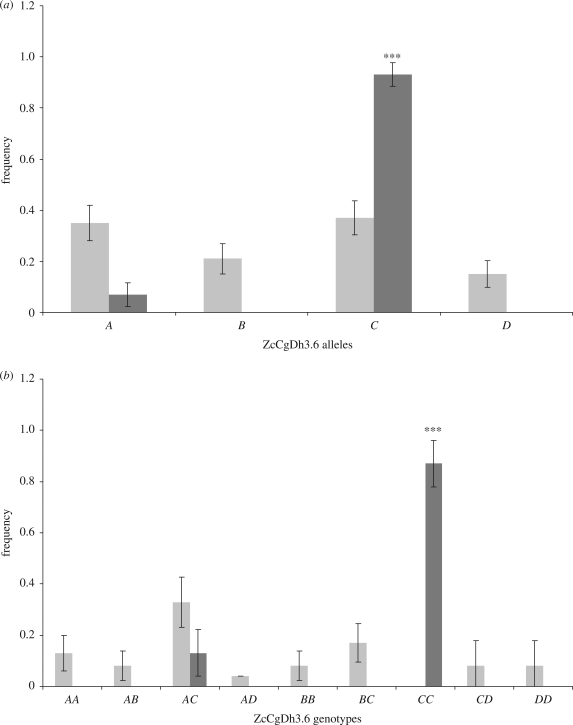
Microsatellite ZcCgDh3.6 composition in NZSL pups with (dark grey columns) and without (light grey columns) hookworm-related anaemia. (*a*) Allele frequencies and (*b*) genotype frequencies. Bars indicate ±s.e. ***Exact *p*-value less than 0.0001. Infected pups bearing allele *C* were 34 times more likely to be anaemic than pups with other genotypes.

We found no significant correlation among loci (mean *r* = −0.05; s.d. = 0.16), suggesting that our study population was not enriched for inbred individuals and that one or more of the markers might be contributing disproportionately. Hookworm-related anaemia was explained significantly by a single locus (ZcCgDh3.6; GLM, *F* = 11.81, d.f. = 22, *p* = 0.0005). None of the other loci showed similar effects. Pups that were homozygous at ZcCgDh3.6 were four times more likely to develop anaemia than heterozygous pups (OR = 4.48; 95% CI: 1.52–13.18), and the observed effect was driven by one of the four alleles present (*C*, 0.54). Pups bearing allele *C* were 34 times more likely to be anaemic than pups with other genotypes (OR = 34; 95% CI: 7.12–162.41, *p* < 0.0001; figure 1; see table S2 in the electronic supplementary material). To investigate this result further, we analysed whether the effect was explained by HCT or PLT values in live pups. We did not detect any significant effect of ZcCgDh3.6 genotype on HCT (*p* > 0.05), but pups with a ZcCgDh3.6-*CC* genotype had significantly lower PLT (mean = 69 944 × 10^6^ l^−1^) than pups with other genotypes at this locus (mean = 243 779 × 10^6^ l^−1^; Mann–Whitney two-tailed test, *U* = 21, *z* = 2.39, *p* < 0.01; see fig. S2 in the electronic supplementary material).

## 4. Discussion

We have analysed a small, but detailed, pathology and genetic dataset in an attempt to discover whether individual levels of genetic variation of NZSL pups influence their susceptibility to hookworms. Based on a previous study that reports HFC for aspects of hookworm infections in a large population of California sea lions (Acevedo-Whitehouse *et al*. 2006), we hypothesized that similar effects would be prevalent and stronger in the NZSL, where inbreeding is highly likely to occur, given its small and declining population (Wilkinson *et al*. 2003)—one of the smallest population sizes recorded for the 15 extant otariid species (IUCN Red List 2009)—and given its highly restricted breeding distribution (Campbell *et al*. 2006). While the number of sampled pups that were available for this study is relatively small, as is often the case for wildlife studies (and particularly for those that focus on endangered species such as the NZSL), the sample represents approximately 10 per cent of annual pup production at Sandy Bay beach, and the number of dead pups analysed represents around 45 per cent of annual pup mortality at that location (Castinel *et al*. 2007*b*). In this sense, sample size is certainly not insignificant, although results should be treated with some caution.

We found that microsatellite allelic diversity was relatively high and individual levels of heterozygosity were close to what would be expected for pups born to unrelated parents (Coulson *et al*. 1998; Amos *et al*. 2001; Aparicio *et al*. 2006), only three of the 39 sampled pups showing levels that might reflect mating between closely related parents (Balloux *et al*. 2004). This was unexpected as previous studies have reported low levels of genetic diversity at the mitochondrial control region and at the MHC class II DQB gene (Lento *et al*. 2003), consistent with the history of the species, which underwent a population bottleneck following intensive exploitation during the nineteenth century (Childerhouse & Gales 1988). One explanation for having found relatively high levels of (microsatellite) genetic variation could relate to the fact that the NZSL displays a rather unusual breeding strategy, in which breeding males hold territories for relatively short periods during the breeding season and move extensively between breeding sites (Robertson *et al*. 2006). It is possible that such a strategy may reduce the occurrence of close-kin mating even in a small and declining population.

Contrary to one of our predictions, there were no differences in the levels of heterozygosity between dead and live NZSL pups, and we failed to find an association between heterozygosity and uncinariasis (hookworm burden, hookworm-related mortality and anaemia; see §2). The lack of association remained even when using measures (IR and HL) that would expectedly increase genetic resolution over those solely based on heterozygosity (Aparicio *et al*. 2006). There are a number of reasons that could account for these results. First, as mentioned earlier, our dataset may be too small, with little variability in the levels of heterozygosity, thereby reducing or eliminating potential HFCs. However, previous studies that used similar small sample sizes have found significant, albeit modest (typically less than 5% variation explained), correlations between parasite loads and measures of inbreeding. For instance, a study found a significant negative relationship between inbreeding and hookworm burden in California sea lion pups at each age class, with only 27–31 individuals sampled per class (Acevedo-Whitehouse 2004).

A second explanation might be that the power of the estimates may be too low to detect inbreeding depression in the NZSL. Correlations between microsatellite heterozygosity and inbreeding coefficient (*f*) are generally low, even when many markers are used (Coltman & Slate 2003; Slate *et al*. 2004). Even so, a relatively large number of studies of natural populations have reported associations between multi-locus heterozygosity and different aspects of fitness, including neonatal survival and parasite load. However, non-significant results are not likely to be reported (Coltman & Slate 2003). Furthermore, most of the cases that report HFCs in wildlife may in effect be due to a subset of loci being linked to genes showing heterozygote advantage (single-locus effects), rather than to inbreeding (Hansson *et al*. 2004). To test whether our dataset included inbred pups, we applied the method proposed by Balloux *et al*. (2004), repeatedly dividing the microsatellites into two even groups, and searched for a correlation in heterozygosity between the groups. The lack of a positive result further suggests that the measures used are not sufficiently sensitive estimators of inbreeding, even though we used a relatively high number of loci (22), compared with the majority of published studies (typically 5–15 markers; Slate *et al*. 2004).

A third, non-exclusive, explanation relates to differences in the virulence of *Uncinaria* spp. between host species. While hookworms are the main cause of mortality for California sea lion pups from San Miguel Island, CA, USA (Lyons *et al*. 2005), bacterial infections and trauma are the main causes of death in the NZSL (Castinel *et al*. 2007*b*). Furthermore, in California sea lions, *Uncinaria* spp. cause a life-threatening pathology known as hookworm enteritis–bacteraemia complex (Spraker *et al*. 2007), which has not been observed in the NZSL (Castinel *et al*. 2007*b*). Recent work has shown that the buccal capsule and oesophageal lengths of *Uncinaria* spp. collected from NZSLs are shorter than have been reported for *Uncinaria* spp. in other pinniped species (Castinel *et al*. 2006). Such differences could reflect disparities in the hookworm's ability to inflict damage. Although all pups were infected with hookworms, these parasites caused the death of only slightly over 50 per cent, and anaemia was observed in an even smaller subset of the pups, suggesting that hookworms are less virulent in the NZSL than in the California sea lion. If heterozygosity influences resistance to hookworms, selection against relatively less heterozygous hosts would expectedly be weaker for less virulent hookworm species.

When testing for the effects of homozygosity at individual loci, it was interesting to find evidence of heterozygote advantage at a single locus (microsatellite ZcCgDh3.6). Pups homozygous at that locus were significantly more likely to develop anaemia than heterozygous pups. An analogous result was observed for California sea lion pups, where hookworm-induced anaemia was also related to homozygosity at a single microsatellite (Acevedo-Whitehouse *et al*. 2006). When analysing our result in more detail, we found that rather than homozygosity at ZcCgDh3.6 *per se*, the detrimental effect was driven by a particular allele (*C*), which was found at a relatively high frequency in the NZSL pups (0.54). Assuming that hookworm-infected pups that did not develop anaemia are similar to anaemic pups in all other respects, then the OR between ZcCgDh3.6-*C* and anaemia status provides a measure of association between this allele and the risk of developing anaemia in infected pups.

While various factors can influence the development of anaemia—including chronic and congenital disease, haemolysis and nutritional deficiencies—based on what has been described of hookworm pathogenesis in otariids (Spraker *et al*. 2007), it is likely that anaemia was caused by blood loss in the NZSL pups. Hookworms attach to the intestinal mucosa to feed on blood, causing epithelial lesions and damaging capillaries (Stanssens *et al*. 1996). For a mechanically strong haemostatic blood clot to form after a capillary vessel is injured, a sufficient number of functional PLT counts and a complete set of coagulation factors are necessary (Nurden & Nurden 2008). Selection on haemostasis is likely as hookworms produce potent inhibitors of coagulation and PLT function (Stanssens *et al*. 1996). Thus, microsatellite ZcCgDh3.6 is likely to be linked to a gene either involved with an aspect of blood clotting or with erythropoiesis. Interestingly, we found that PLT counts were significantly lower in pups with a ZcCgDh3.6*-CC* genotype, but did not detect any significant difference in HCT values between genotypes. This suggests that the identified locus is likely to be in linkage disequilibrium with a gene involved with PLT function or thrombocytopoiesis, both of which are processes susceptible to harmful mutations (Nurden & Nurden 2008) and would probably be under selection. A recently identified mutation in the beta-1-tubulin gene has been associated with macrothrombocytopenia in dogs (Davis *et al*. 2008), and it is plausible that similar functional mutations might also occur in the NZSL. Regrettably, information on otariid genomic sequences is extremely scarce, making it difficult to explore whether the observed linkage is genuine. Future studies on immunogenetics and mapping of this locus in the NZSL and other otariids might provide a means to study this association in more detail.

If ZcCgDh3.6*-C* reflects a functional mutation involved in the PLT function or thrombocytopoiesis, the obvious question that arises is why has a detrimental allele persisted in the NZSL population? One explanation could be related to the population bottleneck that occurred during the nineteenth century (Childerhouse & Gales 1988), resulting in a very small effective population size. Under this demographic model, the NZSL could have had a high frequency of ZcCgDh3.6*-C*, and selective pressures against this allele were not stringent enough to reduce its frequency in ensuing generations. Alternatively, the allele might persist as a balanced polymorphism. Investigating these possibilities was outside the scope of this study, but needs to be addressed in future studies.

In conclusion, we found no evidence of inbreeding in the NZSLs studied, nor did we find any indication that reduced heterozygosity affects NZSL resistance to hookworms at early stages of pup development. However, we found compelling evidence of an association between one of the microsatellites used and the occurrence of hookworm-related anaemia, similar to what has been observed for a closely related species. Investigating anaemia in more detail showed that the microsatellite was associated with PLT counts, suggesting that aspects of haemostasis, particularly thrombocyte-related, may be under selection. Further studies are needed to ascertain the gene(s) linked to the identified marker and whether similar effects on haemostasis are detectable in other otariid species susceptible to hookworms.
